# Increased risk of hip fractures in patients with dementia: a nationwide population-based study

**DOI:** 10.1186/s12883-014-0175-2

**Published:** 2014-09-12

**Authors:** Hao-Kuang Wang, Chao-Ming Hung, Sheng-Hsiang Lin, Yi-Cheng Tai, Kang Lu, Po-Chou Liliang, Chi-Wei Lin, Yi-Che Lee, Pei-Hsuan Fang, Li-Ching Chang, Ying-Chun Li

**Affiliations:** Institute of Clinical Medicine, National Cheng Kung University, Tainan, Taiwan; Department of Neurosurgery, E-Da Hospital, I-Shou University, Kaohsiung, Taiwan; Department of General Surgery, E-Da Hospital, I-Shou University, Kaohsiung, Taiwan; Department of Neurology, E-Da Hospital, I-Shou University, Kaohsiung, Taiwan; Institute of Health Care Management, National Sun Yat-Sen University, Kaohsiung, Taiwan; Department of Family Medicine, E-Da Hospital, I-Shou University, Kaohsiung, Taiwan; Department of Nephrology, E-Da Hospital, I-Shou University, Kaohsiung, Taiwan; Department of Occupational Therapy, I-Shou University, Kaohsiung, Taiwan

**Keywords:** Dementia, Bone Fracture, Hip, Osteoporosis, Retrospective, Cohort

## Abstract

**Background:**

Dementia has been associated with an increased risk of hip fracture. However, little research has been conducted on the impact of dementia on wrist or vertebral fracture development. The aim of this study was to investigate whether dementia is a risk factor for different types of fracture in Taiwan.

**Methods:**

The study sample was drawn from Taiwan’s National Health Insurance Research Database of reimbursement claims, and comprised 1408 patients who visited ambulatory care centers or were hospitalized with a diagnosis of dementia. The comparison group consisted of 7040 randomly selected individuals. Cox proportional hazard regression model was used to examine associations between dementia and the risk of different types of fracture.

**Results:**

During a 3-year follow-up period, 264 patients with dementia (18.75%) and 1098 patients without dementia (15.60%) developed fractures. Dementia was independently associated with increased risk of hip fracture [adjusted hazard ratio (HR) 1.92, 95% CI 1.48–2.49]. Patients with dementia and osteoporosis had the highest risk of developing hip fracture (adjusted HR 2.27, 95% CI 1.28–4.01). Dementia did not increase wrist fracture or vertebral fracture risk when compared to the control group, even in patients with osteoporosis.

**Conclusions:**

Individuals with dementia are at greater risk of developing hip fracture, particularly if they also have osteoporosis. Early mental screening programs and health education should be initiated to decrease disability and dependence in patients with dementia.

## Background

Dementia and fracture are increasingly common problems in the elderly population. About 0.5% of the global population, or more than 35 million people worldwide, have dementia. These figures are expected to increase: the number of people with dementia is set to double in the next 20 years [1,2]. Dementia has been associated with an increased risk of falling and low bone mineral densities (BMDs) [3,4]. Both falling and low BMDs are known risk factors for fracture, and are leading contributors to disability and dependence in patients with dementia [5-8].

The likelihood of a fracture depends on the force conveyed to the bone by a fall, and the strength of the bone [9]. Several recent studies have reported an association between dementia and hip fracture risk [4,9-17]. People with dementia tend to have a high risk of falling, which has been assumed to explain the relationship between dementia and hip fractures [4,9-17]. In addition, osteoporosis is one of the strongest predictors of hip fracture in patients with dementia [4,10,12,16], and wrist and vertebral fractures are common manifestations of osteoporosis [18]. However, little research has been conducted on the impact of dementia on wrist or vertebral fracture development. Nationwide studies from Taiwan, Sweden, Australia, Canada, and Finland show that persons with AD have higher risk of hip fracture in comparison to the general population. However, many of these studies are characterized by problems such as lack of a definitive diagnosis of dementia, while others focus on only hip fractures [9,11,13,15,17]. This makes it difficult to determine the risks different types of fracture in dementia patients, particularly those with osteoporosis. The purpose of this population-based cohort study was to determine whether dementia is a risk factor for different types of fracture in Taiwan.

## Methods

### Database

This retrospective, population-based cohort study used data sourced from Taiwan’s National Health Institute Research Database (NHIRD), which has been described previously [19]. Briefly, the Longitudinal Health Insurance Database (LHID2000) of the National Health Institute (NHI) randomly selected 1 million insured subjects from the NHIRD database, which comprises healthcare data from the medical records of all beneficiaries (more than 99% of the 23.37 million people in Taiwan). The data represent original medical claims for all islanders covered by the NHI program and are distributed by sex, age, or amount of average payroll-related insurance payments. Diagnoses and procedures are coded according to the International Classification of Diseases, Clinical Modification, Ninth Revision (ICD-9-CM) code. Insurance reimbursement claims data used in this study were available from NHIRD for public access, and patient identification has been encoded to ensure confidentiality. The details of database generation, monitoring, and maintenance are published online by the Taiwan NHRI. The study was approved by the NHRI Ethics Review Committee, Taiwan.

### Study sample

**Figure 1:**
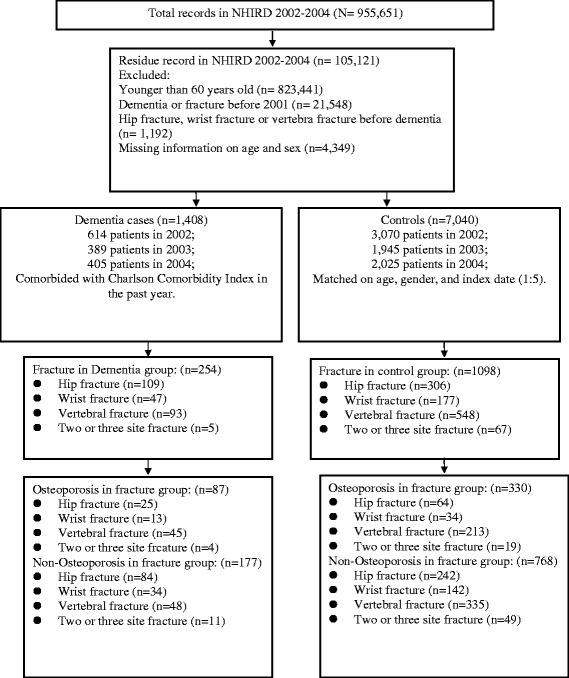
**Flow chart of the study population.**

The study group was composed of patients with dementia [International Classification of Diseases, Ninth Revision, Clinical Modification (ICD-9-CM) codes: 290.0, 290.1, 290.2, 290.3, 290.4, 294.1, 331.0, 331.1, and 331.2] between January 2002 and December 2004 [9]. We assigned the first ambulatory care visits or hospitalizations for receiving a dementia treatment between 2002 and 2004 as the index date. We included only cases that consisted of at least 3 NHI ambulatory-claim records, or 1 inpatient record, in order to increase the validity of dementia diagnosis. The study flow chart is shown in Figure 1. We excluded patients who were younger than 60 years of age. Then, patients diagnosed with dementia before 2001 were excluded. In addition, patients who had hip fracture (ICD-9-CM codes 733.14, 733.15, 820, 821.0, and 821.1), wrist fracture (ICD-9-CM codes 813.4, 813.5, 813.8, and 813.9), or vertebral fracture (ICD-9-CM codes 733.13, 805, and 806) prior to the index use of health care facilities were identified and excluded from the study. A total of 1408 subjects with dementia from 2002 to 2004 were enrolled in this study.

A comparison group was randomly extracted from the records of the remaining patients in the database with identical exclusion criteria. The control group comprised 7040 randomly selected age- and sex-matched individuals without a history of dementia, with 5 comparison subjects for every patient with dementia.

### Main outcome measures

The primary endpoint of this study was the occurrence of hip, wrist, or vertebral fracture as the main diagnosis during a 3-year period from the individuals’ index use of health care. We further calculated different fracture incidence rates between cohorts depending on dementia subtypes. The dementia subtypes were Alzheimer’s Disease (AD) (ICD-9-CM code 331.0) and unspecified dementia (ICD-9-CM codes 290.0 to 290.4, 294.1, and 331.1 to 331.2).

### Statistical analysis

The SAS system (SAS System for Windows, version 9.2, SAS Institute, Inc., Cary, NC, USA) was used to perform the statistical analyses. We compared the distributions of demographic characteristics and selected comorbidities between cases and controls by using Pearson’s chi square test. Comorbidities were included only if they occurred in either the inpatient setting or 2 or more ambulatory care claims recorded 1 year before the index ambulatory care visit. Multivariate Cox proportional hazard regression models were conducted to assess the hazard ratios (HR) with 95% confidence intervals (CI) for risk of fracture after dementia, adjusting for age, sex, and selected comorbidities. In addition to the abovementioned comorbidities, we also adjusted for osteoporosis (ICD-9-CM codes 733.0) and non- osteoporosis in the regression modeling. A *p* value <0.05 was used to assess statistical significance in this study.

## Results

The demographic data of the patients with dementia, and the controls, are shown in Table 1. There were no significant differences between groups in age, gender, and geographic region. Of 8448 people aged ≥60 years enrolled between 2002 and 2004, 1408 patients visited outpatient services or were admitted with a diagnosis of dementia; the 7040 people without dementia were controls. Men comprised 47.66% of patients with dementia. The mean age of the dementia cohort and control cohort was 76.38 ± 7.45 years. Patients with dementia had higher rates of coexisting diseases, including diabetes, dyslipidemia, hypertension, coronary heart disease, heart failure, atrial fibrillation, peripheral vascular disease, cerebrovascular disease, respiratory system disease, peptic ulcer disease, chronic liver disease, chronic kidney disease, rheumatologic disease, and cancer.

**Table 1:** **Comparison of demographic characteristics and comorbidities between patients with and without dementia**

	**Patients with dementia**		**Comparison patients**		
	**(N** ** =1408)**		**(N** ** =7040)**		
**Characteristic**	**N**	**%**	**N**	**%**	**P**
Sex					1.000
Male	671	47.66	3355	47.66	
Female	737	52.34	3685	52.34	
Age, mean ± SD	76.38 ± 7.45		76.38 ± 7.45		1.000
Age					1.000
60-74	270	19.18	1350	19.18	
≥ 75	1138	80.82	5690	80.82	
Geographic region					0.4799
Northern	600	42.61	3017	42.86	
Central	348	24.72	1795	25.50	
Southern	398	28.27	1859	26.41	
Eastern	39	2.77	238	3.38	
Missing	23	1.63	131	1.86	
Comorbidities					
Diabetes	263	18.68	119	1.69	<.0001
Dyslipidemia	193	13.71	94	1.34	<.0001
Hypertension	718	50.99	447	6.35	<.0001
Coronary heart disease	305	21.66	198	2.81	<.0001
Heart failure	65	4.62	52	0.74	<.0001
Atrial fibrillation	19	1.35	17	0.24	<.0001
Peripheral vascular disease	57	4.05	35	0.50	<.0001
Cerebrovascular disease	269	19.11	104	1.48	<.0001
Respiratory disease	238	16.90	148	2.10	<.0001
Peptic ulcer disease	252	17.90	162	2.30	<.0001
Chronic liver disease	141	10.01	66	0.94	<.0001
Chronic kidney disease	95	6.75	42	0.60	<.0001
Rheumatologic disease	43	3.05	20	0.28	<.0001
Cancer	46	3.27	43	0.61	<.0001

*SD*, standard deviation.

During the 3-year follow-up period, 1362 (16.12%) of 8448 patients experienced fractures. Among them, 234 were from the dementia group (15.25% of patients with dementia), and 967 from the control group (12.61% of the controls; Table 2). After adjusting for age, gender, and presence of the coexisting diseases listed above, the hazard ratio (HR) of fracture was 1.34 times greater (95% CI 1.14–1.58) in the dementia group. We further analyzed the HR values according to the different fracture sites (hip, wrist, or vertebral fracture). The HR values obtained within the hip fracture group, wrist fracture group, and vertebral fracture group were 1.92 (95% CI 1.48–2.49, *p* < 0.05), 1.66 (95% CI 0.97–2.55), and 0.88 (95% CI 0.67–1.14), respectively. Table 2 also shows the relative risk of different types of fracture depending on dementia subtype. It is noteworthy that the adjusted HRs for hip fracture in patients with AD was 2.19 (95% CI 1.43–2.88, *p* < 0.05).

**Table 2:** **Fracture risk among sampled patients during the 3**-**year follow**-**up period from index health care utilization** (**N** = **8**,**448**)

	**Patients with dementia N** ** = 1408**		**Alzheimer disease N** ** = 183**		**Unspecific dementia N** ** = 1225**		**Comparison patients N** ** = 7040**	
**Fracture occurrence**	**N.**	**%**	**N.**	**%**	**N.**	**%**	**N.**	**%**
Total	264	18.75	38	20.77	226	18.45	1098	15.60
Crude HR (95% CI)	1.37* (1.19- 1.57)		1.40* (1.18-1.61)		1.36* (1.13-1.67)		1.00	
Adjusted HR (95% CI)	1.34* (1.14- 1.58)		1.37* (1.13-1.59)		1.33* (1.11-1.63)		1.00	
Hip fracture	109	7.74	17	9.29	92	7.51	306	4.35
Crude HR (95% CI)	2.03* (1.63- 2.52)		2.31* (1.57- 2.76)		2.01* (1.49- 2.93)		1.00	
Adjusted HR (95% CI)	1.92* (1.48- 2.49)		2.19* (1.43- 2.88)		1.87* (1.33- 2.83)		1.00	
Wrist fracture	47	3.34	7	3.83	40	3.27	177	2.51
Crude HR (95% CI)	1.43 (0.92- 2.04)		1.55 (0.95- 2.61)		1.16 (0.88- 2.74)		1.00	
Adjusted HR (95% CI)	1.66 (0.97- 2.55)		1.49 (0.98- 2.57)		1.34 (0.91- 2.88)		1.00	
Vertebral fracture	93	6.61	14	7.65	79	6.45	548	7.78
Crude HR (95% CI)	0.99 (0.79- 1.23)		1.00 (0.83- 1.31)		0.91 (0.72- 1.78)		1.00	
Adjusted HR (95% CI)	0.88 (0.67- 1.14)		0.92 (0.69- 1.22)		0.88 (0.62- 1.53)		1.00	

*P < 0.05. HR, hazard ratio; CI, confidence interval. Hazard ratios were calculated with the stratified Cox proportional regression method (stratified by sex, age group, and the year of index health care use) during the 3-year follow-up period. Adjustments were made for Charlson Comorbidity Index in the patients.

Tables 3 and 4 show the relative risk of developing different types of fracture depending on the presence of osteoporosis. The risk of wrist (HR 2.39, 95% CI 0.98–5.80) or vertebral fracture (HR 0.87, 95% CI 0.58–1.30) did not increase in the dementia group compared to the control group, even in patients with osteoporosis. Dementia was significantly associated with higher risks of hip fracture, regardless of age, gender, presence of selected comorbidities, and osteoporosis: HR 2.27 (95% CI 1.28–4.01, *p* < 0.05) with osteoporosis, and HR 1.84 (95% CI 1.37–2.46, *p* < 0.05) without osteoporosis, according to stratified Cox proportional hazard regressions (stratified by sex, age group, and the year of index health care use).

**Table 3:** **Fracture risk among patients with osteoporosis during the 3**-**year follow**-**up period from index health care utilization**

	**Patients with dementia N** = **349**		**Comparison patients N** = **1497**	
**Osteoporosis group**	**N**	**%**	**N.**	**%**
Total				
Osteoporosis	87	24.93	330	22.04
Crude HR (95% CI)	1.33 (1.04- 1.69)*		1.00	
Adjusted HR (95% CI)	1.32 (0.98- 1.78)		1.00	
Hip fracture				
Osteoporosis	25	7.16	64	4.28
Crude HR (95% CI)	1.98 (1.24- 3.15)*		1.00	
Adjusted HR (95% CI)	2.27 (1.28- 4.01)*		1.00	
Wrist fracture				
Osteoporosis	13	3.72	34	2.27
Crude HR (95% CI)	1.85 (0.92- 3.70)		1.00	
Adjusted HR (95% CI)	2.39 (0.98- 5.80)		1.00	
Vertebral fracture				
Osteoporosis	45	12.89	213	14.23
Crude HR (95% CI)	1.07 (0.78- 1.48)		1.00	
Adjusted HR (95% CI)	0.87 (0.58- 1.30)		1.00	

*P < 0.05. HR, hazard ratio; CI, confidence interval. Hazard ratios were calculated with the stratified Cox proportional regression method (stratified by sex, age group, and the year of index health care use) during the 3-year follow-up period. Adjustments were made for Charlson Comorbidity Index in the patients.

**Table 4:** **Fracture risk among patients without osteoporosis during the 3**-**year follow**-**up period from index health care utilization**

	**Patients with dementia N** = **1059**		**Comparison patients N** = **5543**	
**Non-Osteoporosis group**	**N**	**%**	**N.**	**%**
Total				
Non-osteoporosis	177	16.71	768	13.86
Crude HR (95% CI)	1.36 (1.15- 1.60)*		1.00	
Adjusted HR (95% CI)	1.35 (1.11- 1.64)*		1.00	
Hip fracture				
Non-osteoporosis	84	7.93	242	4.37
Crude HR (95% CI)	2.04 (1.59- 2.62)*		1.00	
Adjusted HR (95% CI)	1.84 (1.37- 2.46)*		1.00	
Wrist fracture				
Non-osteoporosis	34	3.21	142	2.56
Crude HR (95% CI)	1.32 (0.87- 1.99)		1.00	
Adjusted HR (95% CI)	1.48 (0.93- 2.43)		1.00	
Vertebral fracture				
Non-osteoporosis	48	4.53	335	6.04
Crude HR (95% CI)	0.86 (0.64- 1.17)		1.00	
Adjusted HR (95% CI)	0.88 (0.62- 1.25)		1.00	

*P < 0.05. HR, hazard ratio; CI, confidence interval. Hazard ratios were calculated with the stratified Cox proportional regression method (stratified by sex, age group, and the year of index health care use) during the 3-year follow-up period. Adjustments were made for Charlson Comorbidity Index in the patients.

## Discussion

The results of this population-based cohort study demonstrate significant associations between dementia and the increased risk of hip fracture, especially in AD. For patients with osteoporosis, the risk of hip fracture was greatest for those individuals with dementia. However, we found no significant association between dementia and the risk of wrist or vertebral fracture, even in patients with osteoporosis.

Previous studies have demonstrated a relationship between dementia and hip fractures [4,9-17]. There are several reasons why patients with dementia may have an increased risk of having a hip fracture.

The association between dementia and fracture risk may be fall-related [4,9,16,20]. People with dementia have decreased brain dopamine activity, which is associated with a decline in motor function. These individuals may also be slower to mobilize, take more steps over a defined distance, and have a greater sway path [21-23]. The impairments of gait and balance are associated with an approximately two-fold increased risk of falls in older people with dementia. In addition, patients with dementia were more likely to have diabetes, hypertension, heart failure, coronary artery disease, and atrial fibrillation within the year preceding the dementia diagnosis. Risk factors such as diabetes and hypertension can impair cerebrovascular autoregulation, leading to an increased prevalence of orthostatic hypotension, vasovagal syncope, or carotid sinus hypersensitivity. Therefore, central autonomic dysfunction contributes to falls in older people with dementia. Psychotropic medication (e.g., antipsychotics, antidepressants, anxiolytics, and sedatives/hypnotics) and cardiovascular medications also have side effects on balance, reaction time, orthostatic hypotension, and extrapyramidal symptoms [4,16]. These side effects increase the risk of falls among patients with dementia. Furthermore, visual impairment is one of the many complications in diabetes mellitus, and is also thought to contribute to the greater risk of falls. These effects help to explain the link between dementia and hip fracture.

Another mechanism that may link dementia to hip fracture is the effect of osteoporosis. Patients with dementia have vitamin D deficiency and lower BMD, and are more likely to have osteoporosis [4,16]. In a meta-analytic study of patients with AD, mean BMD was lower in the AD group compared with those without dementia [10]. In another case–control study, patients with dementia were more likely to have a diagnosis of osteoporosis prior to their fracture than those without dementia (43.8% vs. 37.7%, *p* < 0.05); however, primary preventative treatment rates were low [12]. Patients with dementia also have a lower intake of vitamin D and less sunlight exposure, which is particularly true for nursing home residents [16]. These factors indicate that patients with dementia are more likely to have osteoporosis, and may explain why the highest risk of hip fracture in our study was found for patients with dementia combined with osteoporosis.

According to our result, patients with dementia, especially the osteoporosis group, should be considered for special care to help prevent hip fracture. We suggest that health examinations that include cognitive, behavioral, or psychiatric assessment are needed, since early detection of cognitive dysfunction in patients with dementia may decrease the incidence of hip fracture. Moreover, the risk of hip fracture seems greatest in patients with dementia combined with osteoporosis, suggesting that early discovery and control of osteoporosis could decrease the risk of hip fracture [24,25].

Another important finding in our study was the lack of any consistent associations between dementia and the risk for wrist or vertebral fracture, even in patients with osteoporosis. Fractures of the vertebrae (spine), wrist, and proximal femur (hip), are common types of fractures related to falls and osteoporosis [9,26-30]. Wrist or vertebral fractures are common osteoporotic fractures. However, very few previous studies have evaluated the occurrence of wrist or vertebral fractures in dementia patients. Aggressive conservative management is preferred in most elderly patients, and wrist or vertebral fractures are often asymptomatic. These factors may explain the present findings [18,26-28,30]. Although surgical treatment has been shown to achieve better fracture union for wrist fractures, most elderly patients with wrist fracture are still treated with closed treatment [26-28]. In addition, there is no universally accepted definition of vertebral fracture [18,30]. Vertebral fractures have been assessed using the subjective evaluations of radiologists based on spinal radiographs. Several different methods have been proposed for defining prevalent deformities, and it is unclear which is best [31]. Clinical vertebral fractures may cause back pain, numbness, tingling, weakness, or height loss. However, many vertebral fractures seem to arise without pain, suggesting that current medical practice simply misses most real vertebral fractures [30]. In addition, the adverse effect of wrist or vertebral fractures on most activities of daily living is not as severe as that of hip fractures. Thus, wrist or vertebral fracture are often not recognized and reported, leading to under-diagnosis and under-treatment. Early radiographic diagnosis followed by appropriate therapy would help to prevent underreporting.

The main strength of our study lies in its longitudinal database and large sample population. The problems of insufficient power and the effect of selection biases were minimized by the comprehensive coverage of the NHI system and a large sample size. Our findings match those of a majority of meta-analytic or community-based reports on the relationship between dementia and hip fracture [5,9,11,13,15,17].

There are several limitations of this study. First, the diagnoses of dementia and fracture were made by ICD-9-CM codes. Certain selection biases may exist, and caution must be taken in extrapolating the results. For example, vertebral fracture is often considered to be a “silent disease” until patients seek treatment for other medical problems; therefore, it is possible that it is underreported as a medical condition [30]. Second, it is well known that osteoporosis is a very important risk factor for fracture. This study lacks information regarding the early recognition and treatment of osteoporosis [12]. Third, the NHI database includes only patients who sought treatment for dementia and fracture. Some information was not available, such as data on depression, low educational attainment, and rehabilitation [4,9,16]. Therefore, these variables could not be adjusted for in the analysis. Finally, information on causes of deaths could not be obtained from the NHI database, thereby making an analysis of the mortality ratio related to hip fracture impossible [7,8].

## Conclusions

In conclusion, patients with dementia have a significantly higher risk of hip fracture after adjustments for selective confounding factors such as age, gender, and underlying comorbidities. This study also showed that osteoporosis is an important predictor of hip fracture. Therefore, early detection of cognitive dysfunction and aggressive treatment of osteoporosis in patients with dementia may decrease the incidence of hip fractures. In addition, the dementia group did not show an increased wrist or vertebral fracture risk when compared to the control group, even in patients with osteoporosis. Care must be taken in extrapolating from these results because wrist or vertebral fracture is underreported.

## Abbreviations

AD: Alzheimer disease
APOE4: Apolipoprotein E4
BMDs: Bone mineral densities
CI: Confidence intervals
HR: Hazard ratios
ICD-9-CM: International Classification of Diseases, Clinical Modification, ninth edition
NHIRD: National Health Institute Research Database
NHRI: National Health Research Institute
